# Morphology and Development of Saprophagous Scuttle Fly, *Spiniphora genitalis* Schmitz, 1940 (Diptera: Phoridae) Larvae at Indoor Ambient Temperatures

**DOI:** 10.21315/tlsr2025.36.1.11

**Published:** 2025-03-30

**Authors:** Reena Abd. Rashid, Raja M. Zuha

**Affiliations:** 1 Forensic Science Program, Faculty of Health Sciences, Basement One, Perpustakaan Tun Seri Lanang, Universiti Kebangsaan Malaysia, 43600 Bangi, Selangor, Malaysia; 2 Faculty of Applied Sciences, Universiti Teknologi MARA, 40450 Shah Alam, Selangor, Malaysia; 3 Centre for Insect Systematics, Faculty of Science and Technology, Universiti Kebangsaan Malaysia, 43600 Bangi, Selangor, Malaysia

**Keywords:** Forensic Entomology, Post Mortem Interval, Decomposition, Mollusc Carrion, Larval Instars, Entomologi Forensik, Tempoh Pasca Kematian, Penguraian, Bangkai Siput, Instar Larva

## Abstract

The oriental scuttle fly, *Spiniphora genitalis* Schmitz, 1940 (Diptera: Phoridae), was first discovered from Peninsular Malaysia as a specialised decomposer of mollusc carrion but its occurrences on vertebrate carrion suggest that this species could also be utilised as a forensic indicator. However, the larval morphology and development of *S. genitalis* remain unexplored. In this study, the colony of *S. genitalis* was reared in the laboratory at ambient temperature range (20.0°C–25.5°C) and relative humidity (71.0%–94.5%) using decomposing beef liver as larval food source. Larval body length was measured at every 6 h and 18 h interval until pupariation and subsequently described. The third instar larva of *S. genitalis* can be characterised by its fleshy, but ventrally flattened body with posterior tubular extension. The abdominal segments are covered with tubercles and spinulose formations and interestingly, three distinct larval variations were also detected. The transitions from metapneustic first instar to amphipneustic second and third instar larvae were also described according to the characteristics of cephalopharyngeal skeleton, anterior spiracle and posterior spiracle. The lifecycle of *S. genitalis* reported in this study based on it developmental stages were 23 h–27 h (egg), 162 h–358 h (larva) and 240 h–341 h (pupa). This study established the diagnostic features of *S. genitalis* which can be useful in forensic analysis and as precursors to the ground plan of *Spiniphora* larvae taxonomy. The complete growth of *S. genitalis* larvae on beef liver indicated that the role of this species has been expanded from a specialised mollusc carrion decomposer to a potential forensic indicator.

## Abstract (fr)

**Abstrak:**

Lalat forid *Spiniphora genitalis* Schmitz, 1940 (Diptera: Phoridae) dari timur pertama kali ditemui di Semenanjung Malaysia sebagai pebangkai siput yang khusus tetapi kehadirannya pada bangkai haiwan vertebrata mencadangkan spesies ini berpotensi dijadikan penunjuk forensik. Walau bagaimanapun, sehingga kini masih tiada maklumat mengenai morfologi dan perkembangan *S. genitalis*. Dalam kajian ini, koloni *S. genitalis* dipelihara di makmal pada julat suhu persekitaran (20.0°C–25.5°C) dan kelembapan relatif (71.0%–94.5%) menggunakan hati lembu reput sebagai makanan larva. Panjang badan larva diukur pada setiap selang masa 6 jam dan 18 jam hingga pupariasi dan kemudiannya diperihalkan. Larva *S. genitalis* instar ketiga dapat dicirikan dengan badan yang tebal tetapi leper di bahagian ventral dengan sambungan tiub posterior. Segmen abdomen dilitupi tuberkel dan pembentukan spinulosa, dengan tiga variasi larva dapat dikesan. Peralihan fasa daripada instar pertama metapneustik kepada larva instar kedua dan ketiga amfipneustik juga diperihalkan berdasarkan ciri-ciri rangka sefalofarinks, spirakel anterior dan spirakel posterior larva. Dalam kajian ini, kitaran hayat *S. genitalis* mengikut peringkat perkembangan adalah: 23 jam–27 jam (telur), 162 jam–358 jam (larva) dan 240 jam–341 jam (pupa). Ciri-ciri diagnostik *S. genitalis* dalam kajian ini boleh dijadikan sebagai pelan asas kepada taksonomi larva *Spiniphora* bagi analisis forensik. Keupayaan larva *S. genitalis* untuk melengkapkan kitar hidup pada hati lembu juga menunjukkan peranan dan potensi spesies ini diperkembangkan daripada pebangkai siput yang khusus kepada kepada penunjuk entomologi forensik.

**Highlights**

- *Spiniphora genitalis* larval morphology and instars were described as a diagnostic reference and precursors to the ground plan of *Spiniphora* larvae taxonomy.
- *Spiniphora genitalis* growth on beef liver was recorded in this study which indicated the biology of this species has been expanded from a specialised mollusc carrion decomposer to a potential forensic indicator.
- The developmental duration of *S. genitalis* at ambient temperatures (20.0°C–25.5°C) and relative humidity (71.0%–94.5%) were 23 h–27 h (egg), 162 h–358 h (larva) and 240 h–341 h (pupa).

## INTRODUCTION

*Spiniphora genitalis* Schmitz (Diptera: Phoridae) was first recorded from Peninsular Malaysia inhabiting dead molluscs, *Achatina fulica* Bowdich (Stylommatophora: Achatinidae) (Schmitz 1940). The distribution of this species expands from China (Liu 2001), Sri Lanka (Colyer 1955), Indonesia (Colyer 1955) and Thailand (Disney & Bänziger 2009) of the Oriental region to Fiji of the Australian region (Disney & Bänziger 2009). It is also listed as an immigrant species of Hawaii, USA, possibly introduced by humans (Nishida 2002). Beside *S. genitalis*, there are seven other species of *Spiniphora* from Oriental Region, such as *S. apicalis* Brues, *S. atricostata* Schmitz, *S. experadenya* Disney, *S. nipponensis* Beyer, *S. okinawa* Borgmeier, *S. unicolor* Liu and *S. lawrencei* Disney (Disney & Bänziger 2009; Disney 2023). However, the cosmopolitan species in this group, which is *S. bergenstammi* Mik, still yet to be discovered in Peninsular Malaysia.

The taxonomy of *S. genitalis* mainly relies on the adult male and can be recognised by the yellowish brown thoracic scutum, 4–5 bristles on hind tibia and unusually large hypopygium (Colyer 1955; Disney & Bänziger 2009; Schmitz 1940) while the description of female is covered by Liu (2001). Despite the occurrences of *S. genitalis* in this region, the larval characteristics remain unknown and its developmental duration is still scarce. Members of the genus *Spiniphora* including *S. genitalis* are mainly specialised decomposers of mollusc carrions (Disney 1994) and rarely reported as decomposers of vertebrate carrion such as rabbit carcasses (Zuha *et al*. 2017). However, *S. bergenstammi* has been found on a human corpse (Oliva 2004) suggesting the potential role of this genus as a forensic indicator. For this reason, information regarding taxonomy of the larvae and their developmental duration is important because it can be the primary source of reference when estimating minimum post mortem interval (PMI).

This research was conducted to describe the taxonomic characteristics of *S. genitalis* larvae based on the external morphology of the third instar larva. The third instar is the primary stage of larva development, often used for taxonomic key identification due to the maturity of distinctive morphology features and the prolonged duration compared to previous stages (Sukontason *et al*. 2004). This study also elucidated larval instars based on cephalopharyngeal skeleton, anterior and posterior spiracles as reference to species diagnosis and also instar determination. *Spiniphora genitalis* was chosen as the experimental subject in this study due to its natural abundance at sampling location, Universiti Kebangsaan Malaysia, Bangi, Selangor. The developmental duration of *S. genitalis* at ambient room temperature was also recorded as a preliminary reference for minimum PMI estimation using this species.

## MATERIALS AND METHODS

### Preparation of *S. genitalis* Colony

*Spiniphora genitalis* adults were obtained from three pan traps (28 cm diameter, 3 cm height) baited with decomposed beef liver placed in a 60 mL sterile universal container. The traps were placed approximately 5 m apart on the ground at the forest fringe of Forensic Science Simulation Site, Universiti Kebangsaan Malaysia (UKM), Bangi (2.92°N, 101.79°E). In the laboratory, the adults were preserved in 70% ethanol and subsequently mounted on microscope slides using Canada balsam. Larvae, presumably *S. genitalis*, were reared in the same containers until the adult stage at ambient laboratory temperature. The identity of *S. genitalis* was confirmed based on adult taxonomy according to descriptions by Disney and Bänziger (2009) and reference specimens from Cambridge University Museum of Zoology, UK. Then, they were isolated in a 30 cm (L) × 18 cm (W) × 28 cm (H) acrylic cage and reared until 3rd to 4th generations at ambient temperature (20.0°C–25.5°C) and relative humidity (71.0%–94.5%) with 12:12 h light–dark photoperiods. The adults were supplied with sugar granules, water and beef liver exudates with ratio 1:2:3, respectively.

### Rearing Procedures

The eggs of *S. genitalis* were obtained by introducing 30 g of diced beef liver in a 60 mL sterile plastic container into the colony for a period of 4 h–6 h. The container was then cleared from any adults, sealed with a dry paper towel to avoid further oviposition by other adults, and left inside the 30 cm (L) × 18 cm (W) × 28 cm (H) acrylic cage along with the existing *S. genitalis* adult colony. A data logger (Lascar USB, UK) was placed next to the same acrylic cage to record hourly temperature and relative humidity during the study period. Ambient temperature and relative humidity during the study period are summarised in Table 1.

### Sampling and Data Acquisition

This study was conducted in six replicates. Developmental durations of *S. genitalis* were recorded according to the periodic growth of egg, feeding larva, post-feeding larva and pupa through the observation of the first sample that had transitioned during each sampling occasion (Zuha & Omar 2014). This reflected the minimum time required for the samples to reach certain developmental stage.

To obtain the egg period, samples were observed hourly from oviposition until eclosion. From the onset of the feeding larva period, a total of 10 larvae were randomly sampled twice daily at 6-hours and 18-hours interval post emergence until pupariation. During each sampling occasion, larvae were killed by immersion in hot water (±80°C) for 30 s and measured immediately to avoid preservative and storage effects on the specimens’ length (Matthes *et al*. 2021). Larval body length was measured from the furthest anterior part of the head to the last abdominal segment (Day & Wallman 2008) using SMZ745T stereomicroscope (Nikon, Japan) fitted with a 12-megapixel digital camera (ToupCam, China) and ToupView software (version 3.7). After the measurement, larvae were preserved in 70% ethanol before being subjected to slide mounting in Canada balsam using techniques prescribed by Omar *et al*. (1994). Larval instar periods during feeding larval and post-feeding period were determined by examining the first sample that transitioned during each sampling occasion, using corresponding mounted specimens on slides. After 48 h of the onset of pupariation, the lengths of 5–8 puparia from each study replicate were measured dorsally from the anterior to the posterior end.

## DATA ANALYSIS

The characteristics of *S. genitalis* were described based on the external morphology of the third instar larvae while larval instars were identified based on cephalopharyngeal skeleton, anterior and posterior spiracles (Lee *et al*. 2021). Descriptive analysis of developmental duration (h), larval and puparia morphometry (mm) of *S. genitalis* was performed by using Microsoft Excel 2019 which includes calculations on mean, minimum and maximum values and standard deviation.

## RESULTS

### External Morphology of *S. genitalis* Larva

**Figure 1:**
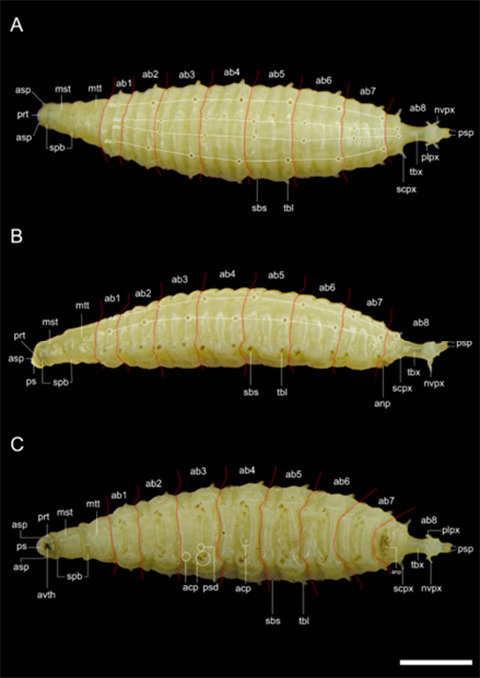
The external morphology of *S. genitalis* third instar larva. (A) View from dorsal; (B) View from lateral; and (C) View from ventral. Red dotted lines show the segmentations of the abdomen based on Keilin (1911) from anterior. *Abbreviations*: ps = pseudocephalon; prt = prothorax; mst = mesothorax; mtt = metathorax; ab1–8 = abdominal segments 1–8; acp = anchor pad; anp = anal pad; asp = anterior spiracle; avth = anteroventral tooth; dls = dorsolateral spine; dsp = dorsal spine; nvpx = near-ventral appendix; plpx = postero-lateral appendix; psd = pseudopod; psp = posterior spiracle; sbs = subconical spine; scpx = subconical appendix; spb = spinose band; tbx = tubular extension. Scale bar = 1 mm.

The third instar larva of *S. genitalis* is fleshy and yellowish, with a narrow anterior end and ventrally flattened body. It is divided into 12 segments, including a posterior tubular extension that bears the posterior spiracles (Fig. 1). The three thoracic segments are located after the pseudocephalon, which is smooth and annulated by spinulose bands. The first and second bands consist of 8 to 9 rows of small single hooked spinules (unicuspid). The third band has 6 to 7 rows of slightly larger unicuspid spinules and sometimes, dorsally, only few of the spinules are double hooked (bicuspid). Ventrally, these spinules are thinner and denser. Anterior spiracles are located near the posterior margin of the first thoracic segment.

**Figure 2:**
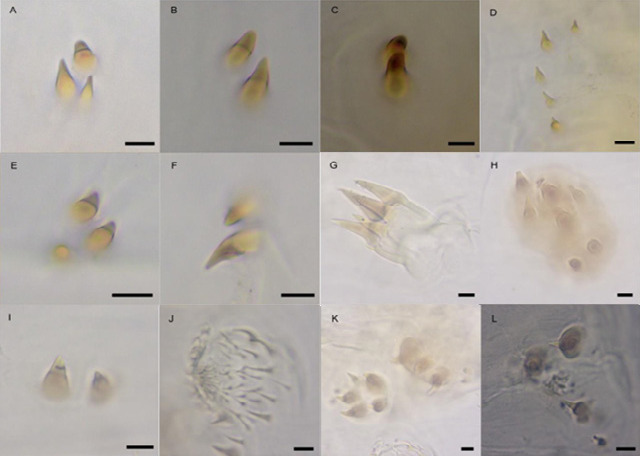
Various spinulose formations on *S. genitalis* third instar larva. (A & B) Subconical spinules with brown tip of dorsal abdominal segments 2–7; (C) Subconical spinules on dorsal abdominal segment 8; (D) Spinulose formation on dorsal abdominal segment 2; (E & F) Spinulose formation on midline of abdominal segment 1 and posterior third of segments 2–7 on each dorsolateral row; (G) Lateral tubercle; (H) Subconical brown spine; (I) Conical spinules on lateral abdominal segments 3–4 (possibly sensory cones); (J) Circular pseudopods on ventral abdominal segments 1–7; and (K & L) Anchor pads adjacent to pseudopods. Scale bar = 0.01 mm.

The abdomen consists of 8 segments and variously covered with spinulose formations and sensilla (Fig. 2). Dorsally, clusters of 2 to 3 subconical spinules with brown tips (Figs. 2A and 2B) are arranged in two rows in the anterior third of abdominal segments 2–8, but on the last segment they are usually blunt (Fig. 2C). The formation of spinules as Fig. 2D is lined almost transversely on the anterior margin of abdominal segment 2 whilst spinulose formations as Figs. 2E and 2F are located on the midline of abdominal segment 1 and posterior third of segments 2–7 on each dorsolateral row. On both sides, each abdominal segment consists of lateral tubercles with 3–5 brown spines at the tip (Fig. 2G). In front of these tubercles are bundles of 3–4 shorter subconical brown spines (Fig. 2H), and usually 1–2 conical spinules (possibly sensory cones) can be found on segments 3 and 4 (Fig. 2I). The formation of locomotory welts or anchor pads on the ventral ridges of abdominal segments 1–7 consists of transverse rows of spinulose bands with unicuspid, bicuspid and tricuspid spinules and circular pseudopods with pale, minute hairs (Fig. 2J). Adjacent to these welts are additional anchor pads comprising two differentiated clusters of spinules (Fig. 2K), of which the subconical shapes and sizes are variable. Spinules formation as Fig. 2L can be found on each side of the ventral grooves of abdominal segments 2–7.

**Figure 3:**
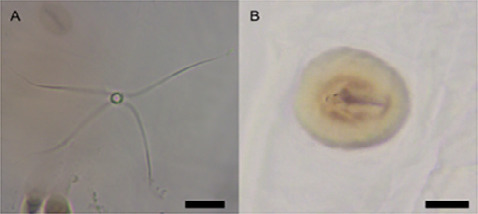
Sensilla on *S. genitalis* third instar larva. (A) Hair sensilla with star-like appearance; (B) Peg sensilla. Scale bar = 0.01 mm.

Hair sensilla with a star-like projection (Fig. 3A) are distributed mainly at the anterior of the first thoracic segment, adjacent to dorsal spinules and at the base of lateral tubercles. A couple of peg sensilla (Fig. 3B) can be located on abdominal segments 1 and 6 in lateral position.

**Figure 4:**
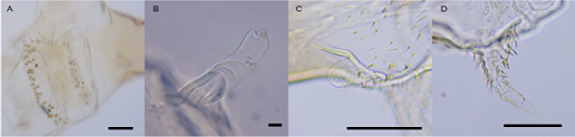
Abdominal segment 8 features of *S. genitalis* third instar larva. (A) Posterior anchor and anal pad; (B) Subconical appendix with apical tuft; (C) Lateral appendix; (D) Near-ventral appendix. Scale bar = 0.01 mm.

The anterior transverse spinulose band beneath abdominal segment 8 is more sclerotised and slightly convex to the anterior, whilst the posterior band is paler with hair-like spinules (Fig. 4A). A lightly sclerotised anal pad is located between these two bands. The abdominal segment 8 bears a pair of laterals, translucent subconical appendices, each with an apical tuft (Fig. 4B). The smooth tubular extension of abdominal segment 8 widens distally holding a pair of lateral appendices (Fig. 4C) pointed forward and another pair of near-ventral appendices which are longer and narrower (Fig. 4D).

**Figure 5:**
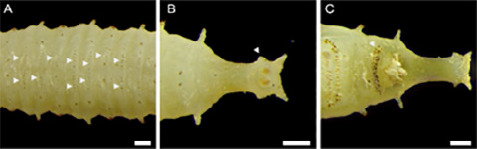
Variations of external larval morphology within *S. genitalis* larvae. (A) Transverse rows of dorsal spinules on abdominal segments 3–6 (arrow); (B) Short lateral appendix (arrow); and (C) Wider ventral spinulose band (arrow). Scale bar = 0.2 mm.

It is important to note that there are three distinct forms of external larval morphology of *S. genitalis* observed in this study. The second group differs from the first group (Fig. 1) by having transverse rows of fine hairs on abdominal segments 3 to 6 (Fig. 5A). The third group displays dorsal spinules on abdominal segments 3 to 6. These spinules and hairs are arranged in combinations of complete or incomplete rows and do not extend beyond the dorsolateral margins where the formations of spinules as in Figs. 2E and 2F are located. The tubular extension and lateral appendices of abdominal segment 8 are also shorter in this group (Fig. 5B) but the ventral spinulose band in front of the anal pad is wider (Fig. 5C). Despite these differences, the ornamentation of various spinules and protuberances of the larval body in these groups are similar.

### Larval Instars of *S. genitalis*

#### First instar

**Figure 6:**
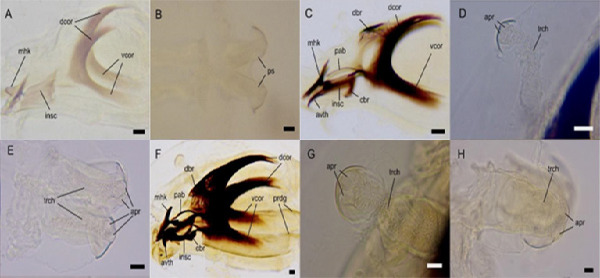
Larval instars of *S. genitalis*. (A) Cephalopharyngeal skeleton of first instar; (B) Posterior spiracles of the first instar; (C) Cephalopharyngeal skeleton of the second instar; (D) Anterior spiracle of the second instar; (E) Posterior spiracles of the second instar; (F) Cephalopharyngeal skeleton of the third instar; (G) Anterior spiracle of the third instar; (H) Posterior spiracle of the third instar. *Abbreviations*: avth = anteroventral tooth; apr = aperture; cbr = cross bridge; dbr = dorsal bridge; dcor = dorsal cornua; insc = intermediate sclerite; mhk = mouth hook; pab = parastomal bar; ps = posterior spiracle; prdg = posterior pharyngeal ridge; trch = trachea; vcor = ventral cornua. Scale bar = 0.01 mm.

In the newly hatched first instar larva, the cephalopharyngeal skeleton consists of anterodorsal mouthhooks (mandibles) with a dorsal projection, connected to the pharyngeal sclerite by an intermediate sclerite (Fig. 6A). The anteroventral intermediate sclerite fused apically forming the median tooth. The pigmented section of dorsal cornua subequal to or slightly shorter than the ventral cornua. Anterior spiracles absent. Posterior spiracles are present but indiscernible in the first instar (Fig. 6B).

#### Second instar

The cephalopharyngeal skeleton in the second instar is more sclerotised than the first instar. The mandibles are visibly developed, each with a strong dorsal projection and slightly curved downward (Fig. 6C). They are articulated with a pair of parastomal bars above the intermediate sclerites. The dorsal projections of intermediate sclerites are connected to the mouthhooks and the median anteroventral tooth curve downwards. The two lateral arms of intermediate sclerite are joined by a cross-bridge which is curved downward. The dorsal bridge is present and has a perforated appearance. Dorsal and ventral cornua subequal in length. Posterior pharyngeal ridge present. A pair of knob-like anterior spiracles present on each side of the first thoracic segment (Fig. 6D). Each posterior spiracle is bilobed and mounted on the tubular extension of the last abdominal segment (Fig. 6E).

#### Third instar

The cephalopharyngeal skeleton of the third instar is similar to the second instar. Still, the median tooth is strongly curved downward (Fig. 6F). Dorsal face of the anterior part of mouthhooks consists of tooth-like sclerites. Dorsal cornua are slightly longer than the pigmented part of the ventral cornua. The ventral pharyngeal ridge is more demarcated than the second instar. Anterior spiracle as Fig. 6G. Posterior spiracle as Fig. 6H but in latter phase the tubular extension of abdominal segment 8 is lightly sclerotised.

### Development of *S. genitalis* Larvae and Pupae at Indoor Ambient Temperatures

The eclosion of *S. genitalis* eggs occurred approximately 23 h–27 h after oviposition. Developmental periods of *S. genitalis* larvae were 24 h–48 h (first instar), 30 h–66 h (second instar) and 72 h–286 h (third instar) (Table 2). Upon entering the post-feeding phase, larvae roamed to a drier area and attached vertically side by side on the interior wall of the rearing container to pupariate. Pupariation was indicated by the gradual sclerotisation of the outer cuticle layer, shortening larval length and followed by the formation of respiratory horns within 48 h. Pupariation period across all replicates was 240 h–341 h. As shown in Fig. 7, the shortest developmental duration from larva to adult was observed in study replicate 2 (402 h) at 21.0°C–24.5°C and relative humidity 71.0%–93.5% whilst the longest was in study replicate 3 (670 h) at 20.0°C–25.5°C and relative humidity 76.0%–94.5%.

The morphometry based on mean larval length according to instars was 1.10 ± 0.12 mm (first instar), 2.83 ± 0.65 mm (second instar) and 6.25 ± 0.88 mm (third instar). Puparia mean length was obtained 48 h after pupariation and sexual size dimorphism was detected between male (5.18 ± 0.37 mm) and female (5.54 ± 0.62 mm). However, the number of pupa collected in each replicate is inadequate (*n* < 10) for inferential statistical analysis.

## DISCUSSION

This research describes the diagnostic features of *S. genitalis* based on the morphology of the third instar larva. Externally, *S. genitalis* larva can be identified by its fleshy, ventrally flattened body and elongated abdominal segment 8. The tubular extension of this segment is sclerotised during the third instar to protect posterior spiracles from damage and blockage while the body remains submerged in viscous decaying media to feed (Rotheray 2019). This feature occurs in a range of aquatic Phoridae larvae such as *Megaselia gregalis* (Meijere), *Megaselia schuitemakeri* (Schmitz) (Disney 1991), *Chonocephalus punctifascia* Borgmeier and *Chonocephalus depressus* Meijere (Ferrar 1987). Despite the similar functional characteristics, the larva of *S. genitalis* is closely identical to *S. bergenstammi* (Ferrar 1987; Keilin 1911; Oliva 2004), but the posterior ventral appendices on abdominal segment 8 are absent in *S. genitalis*. The lateral appendices in *S. genitalis* are shorter with apical tuft but in *S. bergenstammi*, these lateral appendices are covered with short hairs.

*Spiniphora genitalis* can also be characterised by various formations of spinules and tubercles covering the abdominal segments to protect the body from abrasion, natural enemies and to facilitate locomotion (Rotheray 2019). In *S. bergenstammi*, the tubercles are arranged on lateral and dorsolateral rows on abdominal segments 2–7 (Keilin 1911) but in *S. genitalis*, they are only restrained to the lateral sides of abdominal segment 1–7 and dorsolateral rows are replaced by spinules.

The three larval instars of *S. genitalis* were further differentiated based on the taxonomic characteristics of cephalopharyngeal skeleton, anterior spiracle and posterior spiracle. The dorsal projection of mouthhook, which possibly used to break the egg open, is similar to *M. spiracularis* Schmitz (Lee *et al*. 2021) and *S. bergenstammi* (Keilin 1911). Similarly, the transition from the first to the second instar was also distinctively marked by the presence of a dorsal bridge on cephalopharyngeal skeleton. *Spiniphora genitalis* is also devoid of anterior spiracles during the first instar (metapneustic) and later transformed to amphipneustic during the second instar. The third instar can be distinguished from the second instar by having tooth-like sclerites on mouthhook with median anteroventral tooth strongly curved downward and shorter sclerotisation of ventral cornua which are also found in *S. bergenstammi* (Ferrar 1987; Teskey 1981).

The transformation of the cephalopharyngeal skeleton between larval instars in *S. genitalis* is consistent with *S. bergenstammi*. When referring to the detailed illustrations by Keilin (1911), the morphological characteristics of cephalopharyngeal skeleton, anterior and posterior spiracles in *S. bergenstammi* look strikingly similar to *S. genitalis*, and there is no specific indicator to differentiate the two species. These generic features in *S. genitalis* and *S. bergenstammi* larvae suggest that several morphological features are common and can be treated as the ground plan for the taxonomy of genus *Spiniphora*. This, however, depends on the larval morphologies of more than 20 species representative in genus *Spiniphora* which remain unknown. On top of that, the morphological anomalies within *S. genitalis* larvae observed in this study warrant further investigation on whether they are attributed to genetic polymorphism, sexual dimorphism or natural habitat. Contamination of other species within the same colony was unlikely to occur in this study because all newly emerged adults from each sample replicate were strictly examined and identified as *S. genitalis*. Previous observations showed that it is not impossible for the same species in Phoridae to have different larval forms, but the evidence is still inconclusive (Ferrar 1987). For instance, *Chonocephalus heymonsi* Strobbe larvae with elongated hind segment inhabited more liquid parts while those with normal hind segment lived in drier part of the same food source, which it was possibly due to feeding strategies in different larval instars (Disney 1994).

In the current study, the use of light microscopy did not provide adequate and reliable distinguishing characteristics of anterior and posterior spiracles in *S. genitalis*, and therefore we recommend further observation using scanning electron microscopy (SEM) as has been employed on other species of Phoridae. For instance, in the second instar larva of *M. spiracularis*, the anterior spiracles consist of a circular papilliform structure that contains two straight slits (Feng & Liu 2012a). The two-slit appearances of the anterior spiracles were also present in *M. scalaris* (Loew) (Boonchu *et al*. 2004; Sukontason *et al*. 2002), *Dohrniphora cornuta* (Bigot) (Feng & Liu 2012b) and *Diplonevra peregrina* (Wiedemann) (Feng & Liu 2012c) in second and third instar larvae. The posterior spiracles vary across species but generally consist of a pair of spiracular discs bearing two straight slits with posterior spiracular hairs in between like those in *M. spiracularis* (Feng & Liu 2012a; Lee *et al*. 2021) or slightly protruding bearing 2–4 slits in *D. cornuta* and *D. peregrina* (Feng & Liu 2012b; 2012c).

While the external larval morphology being described using third instar larvae, the first until third instars were identified based on cephalopharyngeal skeleton, anterior and posterior spiracles progression. Newly emerged *S. genitalis* larva has an average of 1.10 ± 0.12 mm. The minimum and maximum lengths recorded in this study (0.98 mm–7.76 mm) are almost similar to *M. scalaris* (1.01 mm–7.46 mm) (Yanan *et al*. 2023). In contrast, both species are larger than the maximum length of *M. spiracularis* reared at constant (between 5.93 mm and 6.32 mm) and fluctuating (between 3.7 mm and 4.59 mm) temperatures (Wang *et al*. 2020; Lee *et al*. 2021). The larval size can be proportional to sexual dimorphism which was not explored in this study. Differences in larval size can be attributed to variations in experimental design such as killing and storage method, type of rearing substrate and temperature.

In the current study, all *S. genitalis* larvae was directly measured after fixation in hot water to avoid the preservative effect on the body length because there were certain issues in forensic entomology practice when larval body length that is widely used as reference to indicate age, can be variably affected by preservatives. For instance, the use of 70%–80% ethanol could decrease the larval length to approximately 48 h–72 h after preservation, in contrast to larvae in higher concentrations of ethanol with the least effect on larval size (Bugelli *et al*. 2017; Mathes *et al*. 2021). Additionally, the type of food substrate consumed by the larvae could also produce unpredictable sizes. Typically, a food substrate with high fat content could extend the larval developmental period but might show reduction in larval size, compared to lean liver and meat dietaries (El Hadi Mohamed *et al*. 2021). Another study using *M. scalaris* reared in liver agar diet had a prolonged 7 h–15 h developmental period but marginally decreased mean larval length and weight compared to those provided with lean red meat (Zuha *et al*. 2012). Due to the inconsistent effect of these factors on larval body length, cephalopharyngeal skeleton had been suggested as a more reliable source of indicator for larval age (Zuha 2021) but its growth rate was significantly slower than larval body length (Zuha 2022).

The *S. genitalis* larvae from this study were also successfully developed on decomposing beef liver apart from the reportedly dead molluscs (Disney 1994). In Peninsular Malaysia, *S. genitalis* was the most abundant species found on snail carcasses and the adults emerged 15 days–40 days after the exposure (Beaver 1987). In the current study, the developmental duration for adults to emerge at laboratory temperature (20.0°C–25.5°C) using decomposing beef liver was 17 days–28 days. Its closely related species, *S. bergenstammi*, took approximately 46 days–52 days to complete development from oviposition to adult, but it was reared on beef at a greater range of ambient temperature (10.1°C–25.2°C) (Oliva 2004). There are no published records of *S. genitalis* being reared at fluctuating temperatures until this present study. Comparison with other species of Phoridae such as *M. spiracularis* reared under fluctuating temperatures, indicated a shorter duration of 210 h–284 h (25°C–37°C) compared to *S. genitalis* (Lee *et al*. 2021). When compared with the developmental duration at the nearest constant rearing temperatures for *M. spiracularis* at 22°C (529.6 h) and 25°C (367 h) (Wang *et al*. 2020), *S. genitalis* showed slower developmental duration at 20°C–25.5°C (428 h–696 h). Another study by Yanan *et al*. (2023) using *M.scalaris* reared at 22°C and 25°C recorded developmental durations of 448.2 h and 417.7 h, respectively, which were faster than *S. genitalis* in the current study.

Similar patterns were recorded during egg and larval stages. During egg stage, the eclosion of *M. spiracularis* and *M. scalaris* eggs at 22°C–37°C in previous studies occurred in less than 24 h (Wang *et al*. 2020; Lee *et al*. 2021; Yanan *et al*. 2023), whereas *S. genitalis* eggs in the current study took 23 h–27 h to emerge to first instar larva. As indicated in Table 2, the longest larval period was 384 h in study replicate 3, followed by 258 h (study replicate 5) and 188 h (study replicate 2). The larval developmental period of *S. genitalis* in the current study was longer than previous studies using *M. spiracularis*, which recorded at a range of 48 h–71 h at fluctuating temperatures of 25°C–37°C (Lee *et al*. 2021). This patterns also applies to *M. spiracularis* and *M. scalaris* reared under constant temperature of 22°C and 25°C, at which, each recorded larval period was 367 h–302 h and 268 h–107 h respectively (Wang *et al*. 2020; Yanan *et al*. 2023). Developmental time of *S. genitalis* pupa were almost within the range of *M. spiracularis* and *M. scalaris* (Wang *et al*. 2020; Yanan *et al*. 2023). *Megaselia spiracularis* under fluctuating temperature have shorter pupariation period of 168 h–204 h, almost a half of the period recorded in this study (Lee *et al*. 2021). *Spiniphora genitalis* developmental time in this study is relatively comparable to the development of other Phoridae species reared at constant temperatures than those at fluctuating temperatures. Maximum rearing temperature caused lower relative humidity as recorded by Lee *et al*. (2021) whereby 47%–93% relative humidity was recorded at 25°C–37°C. Compared to this study, more than 70% relative humidity was recorded in this similar to Wang *et al*. (2020) and Yanan *et al*. (2023). Niederegger *et al*. (2010) studied the effect of constant and fluctuating temperature on four forensically important species (*Sarcophaga argyrostoma* (Robineau-Desvoidy) (Diptera: Sarcophagidae), *Calliphora vicina* Robineau-Desvoidy, *Calliphora vomitoria* (Linnaeus), *Lucilia illustris* (Meigen) *and Lucilia sericata* (Meigen) (Diptera: Calliphoridae)) and discovered discrepancy of temperatures can vary considerably and should be interpreted from case to case. However, the effect of fluctuating temperatures on the development of *S. genitalis* can be more natural and biologically relevant than those in controlled conditions.

Additionally, we also observed that pupariation of *S. genitalis* began after the wandering post feeding larvae adhered to the interior wall of the rearing container in rows, vertically side by side with their posterior ends elevated. It might or might not be simultaneously for all larval cohorts, but in this study each group of pupae that adhered to the wall usually consists of about 30 to 40 individuals. Similar behaviour of this species was also reported occurring inside the snail shell (Beaver 1987), and likewise for *S. bergenstammi* inside snail (Keilin 1911) and discarded milk bottles with leftover dregs (Oldroyd 1964).

## CONCLUSION

This study provides the first taxonomic characteristics of *S. genitalis* third instar larva and the distinguishing features of larval instars based on cephalopharyngeal skeleton, anterior and posterior spiracles. However, information regarding larval morphology of genus *Spiniphora* is extremely limited and the only adequate baseline information prior to this study is the meticulous description of cosmopolitan *S. bergenstammi* by Keilin (1911). It is possible that some variations observed in *S. genitalis* can cause uncertainty to its identity and this condition warrants further investigations. In spite of that, the common shared features in both species such as the elongated hind segment and abdominal tubercles can be used as a precursor to establish a ground plan for larval taxonomy of this group*. Spiniphora genitalis* also thrived in decomposing beef liver at room temperature and this served as a baseline reference for future developmental studies which can be extended to forensic application.

## Figures and Tables

**Table 1:** Mean ± SD, ambient temperature range (°C) and relative humidity (%) throughout six study replicates.

Study replicate	Ambient temperature (°C)		Relative humidity (%)	
	
	Range	Mean ± SD	Range	Mean ± SD
1	20.5–24.5	22.9 ± 1.09	71.0–93.5	87.6 ± 5.77
2	21.0–24.5	22.9 ± 1.08	71.0–93.5	88.0 ± 5.76
3	20.0–25.5	22.9 ± 0.87	76.0–94.5	89.6 ± 3.12
4	20.0–24.0	22.8 ± 0.68	78.0–94.5	89.5 ± 2.65
5	21.5–24.5	22.8 ± 0.53	76.0–92.5	87.6 ± 3.05
6	21.5–24.5	22.7 ± 0.51	79.0–92.5	87.9 ± 2.87

**Table 2:** Minimum development duration (h) of *S. genitalis* at ambient temperature and relative humidity based on development stages and study replicates. The third instar larva consists of active feeding and post feeding third instar larvae.

Study replicate	Minimum developmental duration (h)				

	Egg	Larval instar			Pupa

		1	2	3	
1	24	24	66	102	341
2	26	42	48	72	240
3	26	42	30	286	312
4	27	24	48	144	264
5	24	48	66	120	264
6	23	24	48	120	264
